# Non-canonical NF-κB signaling in endothelial cells may enhance synovial inflammation by stimulating angiogenesis

**DOI:** 10.1186/1479-5876-9-S2-P50

**Published:** 2011-11-23

**Authors:** Ae-Ri Noort, Katinka PM van Zoest, Paul Peter Tak, Sander W Tas

**Affiliations:** 1 Division of Clinical Immunology & Rheumatology, Academic Medical Center, University of Amsterdam, The Netherlands

## Background

Pathological angiogenesis can be observed in rheumatoid arthritis (RA) synovial tissue (ST) already in the earliest phase of disease and may be critical in the switch from acute to chronic inflammation. The chemokine CXCL12 is regulated by non-canonical NF-κB signaling and stimulates angiogenesis in endothelial cells (EC). Therefore, the non-canonical NF-κB pathway, with its key mediator NF-κB inducing kinase (NIK), may play an important role in pathological angiogenesis leading to the perpetuation of synovial inflammation in RA.

## Objective

To study the role of non-canonical NF-κB signaling in pathological angiogenesis in RA.

## Materials and methods

ST was obtained via mini-arthroscopy from inflamed joints of RA patients. Expression of NIK and CXCL12 was evaluated using immunohistochemistry and immunofluorescence (IF) microscopy. NIK expression was also studied in Grawitz tumor and breast cancer tissues. Next, the effects of non-canonical NF-κB signaling in EC were studied in vitro using angiogenesis/tube formation assays.

## Results

NIK was highly expressed in vascular structures in RA ST. IF microscopy demonstrated that NIK and CXCL12 were both expressed in high endothelial venules and in EC of small (newly formed) blood vessels. In addition, NIK was also expressed in EC in Grawitz tumor and breast cancer tissues, whereas normal skin EC did not exhibit increased NIK expression. In vitro, EC treated with stimuli that induce non-canonical NF-κB signaling significantly enhanced tube formation 2,5-fold (p<0.05), which could be completely blocked by siRNA targeting NIK.

## Conclusion

NIK is preferentially expressed in EC both in inflamed RA ST and in tumor tissues. In vitro, induction of non-canonical NF-κB signaling resulted in EC activation and enhanced angiogenesis, whereas selective blockade of this pathway using siRNA abrogated these effects. These findings point towards an important role of the non-canonical NF-κB pathway in (pathological) angiogenesis. This could be exploited for the development of new therapies not only for RA, but also for other diseases.

